# Characteristics of polycystic ovary syndrome throughout life

**DOI:** 10.1177/2633494120911038

**Published:** 2020-03-18

**Authors:** Yvonne V. Louwers, Joop S.E. Laven

**Affiliations:** Division of Reproductive Endocrinology and Infertility, Department of Obstetrics and Gynaecology, Erasmus University Medical Centre, 3000 CA, Rotterdam, The Netherlands; Division of Reproductive Endocrinology and Infertility, Department of Obstetrics and Gynaecology, Erasmus University Medical Centre, Rotterdam, The Netherlands

**Keywords:** adolescence, amenorrhea, anovulation, cardiovascular disease, diabetes mellitus, health risks, hyperandrogenism, menopause, oligomenorrhea, polycystic ovarian morphology, polycystic ovary syndrome

## Abstract

Polycystic ovary syndrome is the most common endocrine disorder in women of reproductive age. It is a complex disease in which genetic, endocrine, environmental, and behavioral factors are intertwined, giving rise to a heterogeneous phenotype with reproductive, metabolic, and psychological characteristics. Polycystic ovary syndrome affects women’s health and their quality of life across the life course. During different life stages, the polycystic ovary syndrome phenotype can change, which requires a personalized diagnostic approach and treatment. Polycystic ovary syndrome is a major cause of anovulatory infertility; this disorder is also associated with hirsutism and acne. Diagnosing polycystic ovary syndrome during adolescence is challenging because the polycystic ovary syndrome criteria include normal physiological events that occur during puberty. With increasing age, the syndrome evolves from a reproductive disease to a more metabolic disorder. Along with metabolic disturbances, including insulin resistance and abnormalities of energy expenditure, polycystic ovary syndrome is recognized as a major risk factor for the development of type 2 diabetes and cardiovascular disease in later life. Moreover, there is evidence for familial clustering of endocrine and metabolic features of polycystic ovary syndrome. Environmental factors such as diet and obesity appear to contribute to the phenotype. Treatment should be tailored to the specific concerns and needs of the individual patient and involves restoring fertility, treatment of the metabolic complaints, treatment of androgen excess, and providing endometrial protection. The complexity of the disorder, and the impact on quality of life, requires a timely diagnosis, screening for complications, and management strategies for the long-term health issues associated with polycystic ovary syndrome. The syndrome remains underdiagnosed, and women experience significant delays to diagnosis.

## Introduction

Polycystic ovary syndrome (PCOS) is the most common endocrine disorder in women of reproductive age with a prevalence up to 20%. In conjunction, with its reproductive, metabolic, and psychological features, PCOS poses a significant public health issue.^1^ More than 105 million women aged 15–49 years have been diagnosed with PCOS worldwide.^2^ Moreover, the economic burden of the disorder is considerable; healthcare costs are estimated to be over $4 billion in the United States annually.^2^ Women with PCOS can present with diverse symptoms, including irregular menstrual cycles, excessive hair growth, subfertility, and pregnancy complications. Moreover, PCOS is associated with psychological issues such as anxiety, depression, and disturbed bodily images as well as reduced self-esteem. With increasing age, the syndrome evolves from a reproductive disease to a more metabolic disorder. The metabolic features include insulin resistance, impaired glucose tolerance, type 2 diabetes mellitus (DM2), dyslipidemia, and cardiovascular risk factors.^3^ The diversity of the phenotype emphasizes the need for individual health-risk estimation for a patient with PCOS and her family members to provide appropriate clinical care.

Treatment targeting metabolic abnormalities includes lifestyle changes, medication, and sometimes even bariatric surgery for the prevention and management of obesity. Goals of therapy include fertility, decreased hirsutism and/or alopecia, and providing endometrial protection in order to avoid endometrial cancer. Timely diagnosis and appropriate management strategies of the long-term health sequelae such as DM2, hypertension, and risk factors for cardiovascular disease (CVD) are of paramount importance. Early identification of high-risk patients enables thorough preventive screening and early treatment of adverse complications. At least four clinical phenotypes have been delineated according to current diagnostic criteria. Nevertheless, diagnosing PCOS can be challenging due to variations in clinical features and ethnic differences.^4,5^

## Adolescence

Irregular menstrual cycles as a result of ovulatory dysfunction is a key symptom in PCOS according to the Rotterdam criteria. Among adult women, the menstrual cycle varies from 21 to 35 days, and its duration is on average 28 days. Irregular menses and anovulatory cycles are typical during normal pubertal transition. Hence, it may be difficult to differentiate between normal pubertal development and early signs of PCOS. About half of the menstrual cycles range from 21 to 45 days during the second year after menarche.^6^ Overall, the majority of irregular cycles may be anovulatory 2 years after menarche.^7–10^ By the third postmenarchal year, 95% of cycles are within 21-45 days; however, cycles can remain irregular until the fifth year after menarche.^11,12^ In other words, with increasing gynecological age, that is, the number of years following menarche, fewer females experience menstrual cycles exceeding 45 days.^13^ The onset of regular ovulatory cycles is also associated with age at menarche.^11^ Later age at menarche is associated with a higher chance of remaining oligo-anovulatory during the reproductive life. In girls who will start their period after 13 years of age, half of cycles are ovulatory during the first 4.5 years after menarche.^11^ More than 50% of girls who are oligo-amenorrheic at age 15 remain so during their adult life.^14^ The evidence-based international guidelines state that for adolescent girls who are less than 2 years after menarche and have features suggestive of PCOS, the label ‘increased risk for PCOS’ could be used. Girls considered to be ‘at risk’ for PCOS should be followed longitudinally and reevaluated by 8 years following menarche.^3^

Ascertainment of hyperandrogenism may be difficult. Hyperandrogenism includes clinical (hirsutism, acne, and alopecia) and biochemical (elevated circulating androgen concentrations). Hirsutism is defined as the presence of excessive terminal hair growth in androgen-dependent regions. In the general population, it is estimated that 5–15% of the female population suffers from hirsutism.^15^ Scoring systems, for example, modified Ferriman–Gallwey (mFG) score, can be used to characterize the extent of hirsutism for an individual woman. Ethnicity and life stage may influence the mFG score. Acne is common during puberty. During puberty, androgen levels increase physiologically characterized by the high prevalence up to 80% of acne in teenagers.^16^ Hyperandrogenism should be considered for acne resistant to topical treatment. Several methods to assess for biochemical hyperandrogenism can be used. The most commonly used methods are measurements of bioavailable testosterone and calculated free androgen index (FAI-100 × (total testosterone / SHBG). Direct free testosterone assays should be avoided. Not all women suffering from an androgen excess disorder manifest hirsutism. However, 80–90% of patients with hirsutism will have an androgen excess disorder.^17^ PCOS is a diagnosis of exclusion. Hence, nonclassic congenital adrenal hyperplasia, Cushing’s syndrome, hyperprolactinemia, and androgen-secreting tumors need to be excluded.^5,18^

Polycystic ovary morphology (PCOM) is a common finding identified in women with clinical features typical of PCOS. The 2003 Rotterdam criteria included PCOM in the diagnostic criteria for PCOS.^5^ This introduced milder phenotypes into the PCOS definition with limited data on natural history, prompting calls for phenotype identification and more research.^19^ In adolescents, PCOM can also be detected without any of the accompanying features of PCOS, making it a common normal finding in these girls. Studies report the presence of PCOM in up to 40% of adolescent girls.^20^ Because of the high incidence of PCOM and its nonspecificity in those with a gynecological age of less than 8 years after menarche, ultrasound is not recommended at this life stage for the purposes of diagnosis.^3,4^ However, it has been shown that eumenorrheic women with PCOM have higher androgen levels than eumenorrheic women without PCOM, suggesting they might be at risk for anovulatory menstrual cycles later in life.^21^

A recent study shows that weight gain in early adulthood is significantly associated with symptoms or diagnosis of PCOS.^22^ Weight gain prevention and lifestyle counseling should start from adolescence to attenuate the severity of PCOS. There are only a few studies on emotional well-being or psychosocial features in adolescents with PCOS. These reports indicate that amenorrhea is associated with a lower self-esteem, greater fear of negative appearance, and earlier sexarche.^23^ Hyperandrogenism and acne seem to be associated with poorer body satisfaction. Finally, hirsutism and body mass index (BMI) were negatively associated with several psychological variables. These results suggest that menstrual irregularities might be related to sexarche. Moreover, it should be stressed that the treatment of women with PCOS should notably focus on physical and also on psychological and sexual characteristics during adolescence.^23^

Summarizing, the normal physiologic events that occur during puberty confound the diagnosis of PCOS among adolescent girls. It is strongly recommended to reevaluate adolescent girls within 8 years of menarche or sooner.^3^

## Reproductive age

### Subfertility and pregnancy complications

PCOS accounts for up to 70% of patients with anovulatory subfertility.^24^ Subfertility was 15-fold higher in women reporting PCOS compared with controls independent of BMI. Among women reporting subfertility, fertility hormone treatment was used more often in women reporting PCOS (62% *versus* 33%, respectively) compared with women without PCOS. Nevertheless, IVF use was similar in women with and without PCOS.^25^

Several meta-analyses reviewed the pregnancy and delivery complications in women with PCOS. Most meta-analyses reported an increased risk for gestational diabetes mellitus (GDM), gestational hypertension, pre-eclampsia (PE), and cesarean section in women with PCOS compared with controls.^26–29^ Some studies report a higher risk for miscarriage. However, the independent influence of PCOS in miscarriage is difficult to determine, and overall, it appears likely that other factors, such as fertility treatment, obesity, and age, significantly contribute to miscarriage in PCOS.^30^ Women with PCOS have a four-fold increased risk of developing gestational diabetes compared with the reference group of pregnant women without PCOS. Moreover, the children born from mothers diagnosed with PCOS seem nearly 4 times more often small for gestational age.^31^ The association of PCOS with these outcomes is worsened in hyperandrogenic and obese PCOS phenotypes.^29,30,32^ Moreover, the prevalence of the adverse obstetric and neonatal outcomes differed by geographical continent.^30^ PCOS women from Africa seem to be at increased risk for miscarriage, women from Europe for gestational diabetes, women from America for gestational hypertension, and women from Asia for PE and induction of labor.^30^ Timely surveillance during pregnancy in women with PCOS and identification of high-risk groups improve preventive strategies and management.^29,30^ No published studies have characterized postpartum depression among women with PCOS.

### Obesity and dyslipidemia

The close relation between elevated BMI levels and PCOS is obvious considering the fact that PCOS is associated with overweight and obesity (33–88%).^33^ However, whether obese patients are predisposed to PCOS or whether they are obese because of their PCOS status is continuously debated.^34^ Current evidence suggests that obesity is a modifying rather than a causal factor for PCOS. Indeed, it has been shown that the incidence of PCOS among different BMI groups was quite similar.^35^ Hence, it seems that obesity aggravates the reproductive and metabolic phenotype of PCOS.^35,36^

Obesity increases insulin resistance and the resulting hyperinsulinemia, which in turn increases adipogenesis and decreases lipolysis. Obesity also sensitizes thecal cells to luteinizing hormone (LH) stimulation, resulting in functional ovarian hyperandrogenism. Moreover, obesity affects inflammatory adipokines, which, in turn, increases insulin resistance and adipogenesis.^37^ Lifestyle intervention, preferably including diet, exercise, and behavioral strategies, should be recommended in overweight or obese women with PCOS to effectively reduce weight, central obesity, and insulin resistance.^38^ Appropriate first-line treatment for patients with PCOS during their reproductive age is lifestyle modification.^3,39^

A meta-analyses including 30 studies with the mean age of women <45 years found higher mean serum low-density lipoprotein (LDL) cholesterol (LDL-C), non–high-density lipoprotein (HDL) cholesterol (non-HDL-C), and triglyceride (TG) levels and lower HDL-C levels in women with PCOS compared with control women.^40^ It is suggested that PCOS constitutes an increased risk for dyslipidemia associated with obesity.^41^ Although few studies examine the prevalence of dyslipidemia in older women with PCOS, available evidence suggests that dyslipidemia is common in young women with PCOS and likely persists beyond menopause.^42^

### Insulin resistance and type 2 diabetes

PCOS is associated with insulin resistance and hyperinsulinemia.^43,44^ Women with PCOS had increased prevalence of impaired glucose tolerance and DM2 independently of BMI.^45^ In a Danish registry study, the risk of DM2 was 4 times greater among women with PCOS. Furthermore, DM2 was diagnosed 4 years earlier in women with PCOS compared with unaffected control women.^46^ PCOS is associated with impaired glucose tolerance in up to 30% and DM2 in up to 10% of women with PCOS.^47^ A 10-year follow-up study found that the age-standardized prevalence of DM2 in women with PCOS in their 40s or 50s is 40%, that is, 6.8 times higher than that of the general female population of a similar age.^48^ The prevalence of DM2 in PCOS continues to increase during the late reproductive years.^48,49^ Glycemic status should be assessed (using an oral glucose tolerance test [OGTT], fasting plasma glucose, or HbA1c) at baseline in all women with PCOS and should be repeated every 1–3 years depending on other individual risk factors for diabetes present.^3^

### Metabolic syndrome

Metabolic syndrome (MetS) is a cluster of metabolic disturbances, including central obesity, hyperglycemia and insulin resistance, dyslipidemia, and hypertension. Given the association of PCOS with many of these individual components, it is not surprising that meta-analyses have shown a more than twofold increased risk of MetS in women with PCOS.^45,50^ It is suggested that the association of MetS with PCOS may be androgen driven.^51^ Interestingly, studies in relatives of PCOS women observed an increased prevalence of MetS in fathers and sisters, an increased prevalence of hypertension in fathers, sisters, and brothers as well as an increased prevalence of dyslipidemia in fathers of women diagnosed with PCOS.^52^ This suggests a clustering of metabolic risk in families of women with PCOS, thereby increasing their risk for long-term adverse health sequelae.

### Psychosocial features

Depression and anxiety commonly occur among women with PCOS. Depression scores are increased in up to 40% of women with PCOS independent of BMI; both clinical and community subjects are affected.^53,54^ The mechanisms and etiologies of depression and anxiety remain to be clarified; the effects of treatment regimens are unclear. The prevalence of psychosexual dysfunction in PCOS seems also increased and varies from 13.3% to 62.5%, which appears to be higher than the prevalence in the general population.^55–57^ Although individual studies observe mixed results, a recent systematic review and a meta-analysis suggest an increased prevalence of eating disorders and disordered eating in women with PCOS. In addition, women with PCOS often have more risk factors for eating disorders such as obesity, depression, anxiety, low self-esteem, and poor body image.^58^

In women with PCOS, sexual function and feelings of sexual attractiveness are impaired. The findings imply that sexual function, sexual satisfaction, and psychosocial functioning need to be part of every clinical assessment of women with PCOS.^59^ Health professionals should be aware of the high incidence and severity of psychological issues as well as sexual function and should facilitate appropriate referral and preventive strategies. This should be assessed as part of standard care in PCOS.^3^

## Menopause

Menopause is a life stage which occurs on average at 51 years of age. The natural history of PCOS during the perimenopausal years is unknown. Longitudinal natural history studies defining the postmenopausal phenotypes of PCOS are limited. Therefore, assessment and diagnosis of PCOS at this life stage leads to confusion for health professionals in terms of screening recommendations for long-term health risks.^3^ The three key features of the PCOS phenotype seem to be subject to changes over time, which naturally occur with increasing age.^60^ Aging in patients having PCOS is similarly associated with a loss of follicles and, subsequently, the disappearance of PCOM.^60^ The menstrual cycle length shortens with increasing age as a normal physiological phenomenon. In patients suffering from PCOS, a similar shortening is noticed over time, and a substantial number of these women become eumenorrheic. As some studies showed that menopause might be occurring later on in life in women with PCOS, regular ovulatory cycles might be more frequent toward the end of their reproductive years.^60,61^ The fact that women with PCOS might be entering menopause later in life compared with those who are not suffering from the syndrome seems mainly to be due to a selective enrichment of menopause postponing genetic variants in women with PCOS.^62^

However, postmenopausal persistence of PCOS could be considered likely with continuing evidence of hyperandrogenism after menopause. Similarly, if there is a past diagnosis of PCOS, a long-term history of hyperandrogenism, irregular menstrual cycles, and/or PCOM, during the reproductive years, a diagnosis of PCOS after menopause could be considered.^3^

### Metabolic syndrome

Several studies have observed a higher prevalence of MetS in older women with presumed PCOS diagnosis compared with control women.^51^ Some of these studies also showed a higher prevalence of MetS in the hyperandrogenic PCOS phenotype compared with the nonhyperandrogenic phenotype.^63^ Women with presumed PCOS who did not have MetS at baseline had higher rates of MetS during follow-up of 12 years (incidence rates: PCOS: 3.57 *versus* control: 2.26). This difference was not significant after adjustment for confounders, including BMI, suggesting that women with PCOS who have not developed MetS in their premenopausal years may represent a lower-risk group.^51,64^

### Cardiovascular disease

CVD is one of the leading causes of death in women. Obviously, the presence of factors further increasing CVD risk will have significant public health impact. Postmenopausal women in the later decades of life seem to have the highest risk of CVD; however, some studies have shown the presence of CVD risk factors in PCOS women in early adulthood. In the Australian Longitudinal Study on Women’s Health, a community-based cohort, women with self-reported PCOS had a higher prevalence of hypertension than control women independently of BMI (ages 28–33 years; 5.1% *versus* 1.0%, respectively).^65^ However, other studies showed that after correction for BMI and the presence of DM2, this significant effect becomes smaller with increasing age.^66,67^

No statistical differences were found between PCOS and non-PCOS women in myocardial infarction, stroke, CVD-related death, and coronary artery/heart disease in a meta-analysis.^3,68^ Also, the risk for angina or myocardial infarction between women with and without PCOS did not differ.^68^ However, these findings should be interpreted with caution given the methodological and reporting limitations combined with the small sample sizes of these observational studies. Furthermore, most studies included women with a relatively young age, which limits the interpretation of the available data.^3^ Moreover, the impact of hyperandrogenism, assessed around perimenopausal transition, on cardiovascular outcome in women currently between 70 and 80 years of age showed no relationship between androgens and CVD risk.^66^ In a subpopulation with PCOS, they recorded a similar incidence of CVD, stroke, and coronary heart disease compared with age- and BMI-matched women without PCOS.^66^ A large hospital-based Danish population study did report an increased event rate of CVD in PCOS compared with controls.^67^ They included hospital-referred PCOS patients and compared them with population-based controls, which might have caused ascertainment bias. Also, their definition of CVD was very broad, including both prevalent and incident events. The only prospectively designed study with sufficiently long follow-up was published by a Swedish group. This study did not report an increased incidence of myocardial infarction, stroke, or ischemic heart disease in women with PCOS compared with age-matched controls without PCOS.^69^

As stated in this article, metabolic syndrome and CVD risk factors are increased in PCOS. Cardiovascular health, however, needs to be considered. Data on clinical cardiovascular events are limited. Consequently, overall CVD risk and optimal screening for additional risk factors remain highly controversial.^3^ CVD risk in women with PCOS remains unclear pending high-quality studies; however, prevalence of CVD risk factors is increased, warranting consideration of screening is needed.^3^ Recommendations in terms of the exact age at which the first screening should take place or the frequency of screening have not yet been determined. Moreover, as the diagnostic process accounts for only a minor part of the total healthcare costs for PCOS, screening and early treatment will most likely reduce these healthcare costs.

## Conclusions

PCOS is a complex disease in which genetic, endocrine, environmental, and behavioral factors are intertwined, giving rise to a heterogeneous phenotype with reproductive, metabolic, and psychological characteristics that affect women’s health and quality of life across the life course. Curiously, as women age, the PCOS phenotype evolves with amelioration of clinical features. An individualized approach to diagnosis and treatment benefits all women with PCOS.
